# Skin lesions associated with the use of N95 respirators among health professionals in Brazil in 2020 *

**DOI:** 10.1590/1518-8345.5937.3762

**Published:** 2023-03-27

**Authors:** Elucir Gir, Ana Cristina de Oliveira e Silva, Karlla Antonieta Amorim Caetano, Mayra Gonçalves Menegueti, Maria Girlane Sousa Albuquerque Brandão, Simon Ching Lam, Renata Karina Reis, Silmara Elaine Malagutti Toffano, Fernanda Maria Vieira Pereira-Ávila, Soraia Assad Nasbine Rabeh

**Affiliations:** 1 Universidade de São Paulo, Escola de Enfermagem de Ribeirão Preto, Centro Colaborador da OPAS/OMS para o Desenvolvimento da Pesquisa em Enfermagem, Ribeirão Preto, SP, Brasil; 2 Universidade Federal da Paraíba, Departamento de Enfermagem Clínica, João Pessoa, PB, Brasil; 3 Universidade Federal de Goiás, Faculdade de Enfermagem, Goiânia, GO, Brasil; 4 Tung Wah College, School of Nursing, Hong Kong SAR, China; 5 Universidade Federal do Triângulo Mineiro, Departamento de Enfermagem, Uberaba, MG, Brasil; 6 Universidade Federal Fluminense, Departamento de Enfermagem, Rio das Ostras, RJ, Brasil

**Keywords:** Personal Protective Equipment, N95 Respirators, Skin, Health Personnel, Pandemics, Coronavirus, Equipo de Protección Personal, Respiradores N95, Piel, Personal de Salud, Pandemias, Coronavirus, Equipamento de Proteção Individual, Respiradores N95, Pele, Pessoal de Saúde, Pandemias, Coronavírus

## Abstract

**Objective:**

to investigate the prevalence of skin lesions and factors associated with the use of N95 respirators among health professionals in Brazil.

**Method:**

cross-sectional study conducted with 11,368 health professionals using a respondent-driven sampling method adapted for online environments. Univariate and multivariate analyses were performed to investigate the association between the “skin lesions with the use of N95 respirators” variable and gender, professional category, workplace, training, COVID-19 diagnosis, and availability of sufficient and high-quality Personal Protective Equipment.

**Results:**

the prevalence of skin lesions was 61.8%. Women were 1.203 times (95% CI: 1.154-1.255) more likely to develop a lesion than men. The chances of skin lesions in psychologists (PR=0.805; 95% CI: 0.678-0.956) and dentists (PR=0.884; 95% CI: 0.788-0.992), were lower when compared to Nursing professionals. Professionals with a positive COVID-19 diagnosis and working in the Intensive Care Unit have an increased chance of presenting skin lesions (PR=1.074; 95% CI: 1.042-1.107); (PR=1.203; 95% CI: 1.168-1.241), respectively.

**Conclusion:**

the prevalence of skin lesions caused by the use of N95 respirators was 61.8% and was associated with female gender, professional category, workplace, training, COVID-19 diagnosis, and availability of sufficient and highquality Personal Protective Equipment.

## Abstract (es)

**Objetivo:**

investigar la prevalencia de lesiones cutáneas y factores asociados con el uso de respiradores N95 en profesionales de la salud de Brasil.

**Método:**

estudio transversal realizado con 11.368 profesionales de la salud en el que se empleó un método de muestreo dirigido por encuestados adaptado para entornos en línea. Se ejecutaron análisis univariados y multivariados para investigar la asociación entre la variable “lesiones cutáneas por utilizar respiradores N95” y sexo, categoría profesional, lugar de trabajo, capacitación, diagnóstico de COVID-19 y disponibilidad de suficiente provisión de Equipo de Protección Personal de buena calidad.

**Resultados:**

la prevalencia de lesiones cutáneas fue del 61,8%. Las mujeres fueron 1,203 veces (IC 95%: 1,154-1,255) más propensas a desarrollar lesiones que los hombres. La probabilidad de lesiones cutáneas en psicólogos (RP=0,805; IC 95%: 0,678-0,956) y dentistas (RP=0,884; IC 95%: 0,788-0,992) fue menor en comparación con la encontrada en los profesionales de Enfermería. Los profesionales con diagnóstico positivo de COVID-19 y que trabajan en la Unidad de Cuidados Intensivos son más propensos a sufrir lesiones cutáneas (RP=1,074; IC 95%: 1,042-1,107); (RP=1,203; IC 95%: 1,168-1,241), respectivamente,

**Conclusión:**

la prevalencia de lesiones cutáneas causadas por utilizar respiradores N95 fue del 61,8% y estuvo asociada al sexo femenino, a la categoría profesional, al lugar de trabajo, a la capacitación, a diagnóstico de COVID-19 y a la disponibilidad de suficiente provisión de Equipo de Protección Personal de buena calidad.

## Abstract (pt)

**Objetivo:**

investigar a prevalência de lesões de pele e fatores associados ao uso de respiradores N95 entre profissionais de saúde no Brasil.

**Método:**

estudo transversal realizado com 11.368 profissionais de saúde por meio de um método de amostragem dirigido por respondentes adaptado para ambientes online. Análises univariadas e multivariadas foram realizadas para investigar a associação entre a variável “lesão de pele com uso de respirador N95” e sexo, categoria profissional, local de trabalho, treinamento, diagnóstico de COVID-19 e disponibilidade suficiente de equipamentos de proteção individual de qualidade.

**Resultados:**

a prevalência de lesões cutâneas foi de 61,8%. As mulheres foram 1,203 vezes (IC 95%: 1.154-1.255) mais propensas a desenvolver uma lesão do que os homens. As chances de lesão de pele em psicólogos (RP=0,805; IC 95%: 0,678-0,956) e dentistas (RP=0,884; IC 95%: 0,788-0,992) foram menores quando comparados aos profissionais de Enfermagem. Profissionais com diagnóstico positivo para COVID-19 e que trabalham em Unidade de Cuidados Intensivos têm maior chance de apresentar lesões de pele (RP=1,074; IC 95%: 1,042-1,107); (RP=1,203; IC 95%: 1,168- 1,241), respectivamente.

**Conclusão:**

a prevalência de lesões de pele causadas pelo uso do respirador N95 foi de 61,8% e esteve associada ao sexo feminino, categoria profissional, local de trabalho, treinamento, diagnóstico de COVID-19 e disponibilidade suficiente de equipamentos de proteção individual de qualidade.

**Highlights**

(1) The overall prevalence of skin lesions was 61.8%.

(2) The most affected professional category was Nursing.

(3) Women were more likely to develop skin lesions than men.

## Introduction

The coronavirus disease (COVID-19) caused by the 2019 novel coronavirus, Severe Acute Respiratory Syndrome Coronavirus 2 (SARS-CoV-2), is a severe respiratory infection that spreads primarily through saliva droplets or nose discharges when an infected person coughs or sneezes. SARS-CoV-2 rapid and wide spread prompted the World Health Organization (WHO) to declare a COVID-19 pandemic in February 11 ^th^, 2020 ^( 1)^ . Since then, many healthcare professionals around the world have worked on the front lines against the disease, providing care to patients suspected or confirmed to be infected and, as a consequence, they became a population at a high risk of infection themselves ^( 2)^ . 

To minimize the risk of COVID-19 exposure to and contagion, health professionals use Personal Protective Equipment (PPE) during their workday, particularly, N95 respirators. These respirators [or Filtering Face Piece (FFP2), as named in Europe] are filtering face masks to prevent spread of droplets and potential airborne infectious diseases, which are recommended internationally due to their filtering efficiency ^( 3)^ . Regardless of the shapes or designs of such respirators, they are usually of the “tight” half-face-piece type, and their reliability depends on the wearer’s fit and seal ^( 4- 5)^ . 

The Centers for Disease Control and Prevention (CDC) conducted a laboratory performance evaluation and indicated that the mean penetration by ambient aerosol was found to be 33% in ill-fitting respirators, when compared to 4% in well-fitting ones ^( 6)^ . As a consequence, in order to minimize exposure of the airways and reduce the transmission risk, healthcare professionals should choose a well-fitted respirator (evaluated by a quantitative fit test ^( 4)^ , and adjust the N95 respirator seal ^( 7)^ so that it is in firm contact with the skin ^( 8)^ . Due to the need for a tight seal and proper fit to the face skin, N95 respirators present a particularly high risk of causing skin injuries such as indentations, lacerations, post-inflammatory hyperpigmentation, ulceration, crusting and erythema. Worse lesions can be caused by the use of an N95 respirator, mainly due to friction, excessive pressure on the face skin, and accumulation of moisture ^( 9- 11)^ . 

The increase in the number of health professionals who have had skin lesions caused by the use of medical devices is both alarming and worrying. Such injuries can be the gateway to infections by bacteria, fungi and viruses, including SARS-CoV-2, in addition to promoting pain, discomfort and scarring, as well as impacting on the quality of the care provided to the patients ^( 12)^ , even when N95 respirators are used properly. 

As the use of N95 respirators is mandatory for the safety of COVID-19 front-line care providers in aerosol-generating procedures, it is important to identify the association of skin lesions with the use of such devices, explore characteristics related to the lesions, and identify the most vulnerable anatomical locations and professional categories ^( 13- 16)^ . 

A cross-sectional survey conducted among health professionals who care for COVID-19 patients identified a mean of 2.4 skin lesions per professional ^( 8)^ . However, this study was developed in only one Brazilian state, not representative of the country’s population. 

To the present day, there are no national and population-based studies in the literature describing the prevalence of skin lesions among health professionals using N95 respirators and the variables associated with occurrence of this event.

This study aimed at investigating the prevalence of skin lesions and factors associated with the use of N95 respirators among health professionals resorting to a nation-wide and population-based sample.

## Method

### Study design

A cross-sectional study.

### Participants and selection criteria

Invitations were sent to 12,086 health professionals who work in direct patient care at different health care levels in different regions, including North, Northeast, Midwest, Southeast, and South of major cities and towns. The study included 11,368 healthcare professionals (including but not limited to nurses, physicians, physiotherapists, psychologists, occupational therapists, etc.) who have used N95 respirators during their clinical duties. Students attending any healthcare discipline and performing clinical practices were excluded. This study followed the recommendations set forth in the Checklist for Reporting Results of Internet E-Surveys (CHERRIES) ^( 17)^ . 

### Measurements

The questionnaires included variables related to demographic characteristics, professional category, the type of care provided by the professionals, access to PPE, use of N95 respirators, and presence of skin changes resulting from the use of respirators, such as hyperemia (increased local blood circulation, promoting non- blanchable redness) ^( 18)^ , lesions (skin loss in its partial thickness with exposure of the dermis) ^( 18)^ , itching (an unpleasant sensation in the skin that provokes the desire to scratch) ^( 19)^ , dryness (thickening of the *stratum corneum*, which occurs due to low epidermal aqueous content) ^( 20)^ and blisters/bubbles (closed or open bubbles, filled with serous or serohematic fluid) ^( 18)^ . 

The questionnaires were sent to 15 evaluators. The experts filled in an instrument that contained general assessment items (adequacy and applicability), items that assessed the instrument’s coherence and adequacy to the research objectives, items to assess scientific accuracy and the instrument’s content, and language assessment items (adequacy, clarity, objectivity). The Content Validity Index (CVI) was used to verify content validity, and the I-CVI (Item-level Content Validity Index) was calculated for each item in the instrument, as well as the global CVI. The instrument was considered valid, as all the items evaluated obtained CVI values above 0.85. In the general assessment, the CVI reached a mean value of 0.96.

A pilot study was carried out with 27 healthcare professionals, where the participants were contacted through social media apps. Subsequently, the respondents were invited to send feedback or comments on the survey via WhatsApp®. The pilot study aimed at verifying whether online filling-in would be adequate, as well as whether the items were understandable and easy to answer. All items were considered valid and understandable by the professionals who answered the survey. 

Data collection was initiated after the pilot study and a link was sent to access the Free and Informed Consent Form (FICF), followed by the survey. The completed instruments were hosted on the SurveyMonkey® platform, which only allowed one submission of the forms per Internet Protocol (IP) address, providing some security to the information collected. 

All the information was self-reported.

### Data collection

A study of the online survey type was conducted throughout Brazil between October and December 2020. The professionals were recruited using a Respondent-Driven Sampling (RDS) method adapted to the virtual environment.

In this method, the participants are encouraged to recruit other subjects in the same category as their own, through social networks such as WhatsApp® and Instagram®.

For this study, researchers from all the Brazilian regions were selected, who were responsible for assisting in the selection of research leaders; 47 were selected, at least one from each Brazilian state. All of them underwent a four-hour pre-training session on how to conduct an online survey in the COVID-19 pandemic context and also on the questionnaire to be used. Each leader made 10 recruiting nominations, each of them indicated 10 participants, and so on. Each recruit was duly interviewed and, after the interviews, they also underwent training. For this research, 280 collectors were trained and 45 training sessions were carried out. Each researcher identified health professionals that met the study inclusion criteria (being a health professional, providing direct patient care, and using N95 respirators), and the subsequent participants were identified from the first professionals selected. Each recruiter should record in an Excel spreadsheet the number of participants they invited and how many individuals were recruited by each guest, and so on.

### Data treatment and analysis

The data were exported and analyzed using the R statistical software, version 4.0.4. The outcome variable was “changes in the skin related to the use of N95 respirators”. The independent variables were as follows: gender, region, professional category, working in an Intensive Care Unit (ICU), working in a field hospital, having a positive COVID-19 diagnosis, having received training on COVID-19, and perception of adequate PPE supply and quality.

Descriptive analyses of the categorical variables were performed. A univariate analysis was performed to verify the variables that were previously associated with the outcome. From these results, a screening was conducted to identify and select all variables whose p-values associated with the estimates of the coefficients of these variables were equal to or less than 0.20. A multivariable logistic regression analysis was performed using the stepwise method, generating Odds Ratio (OR) values and the respective 95% confidence intervals (95% CIs).

### Ethical aspects

The study was conducted in compliance with all ethical precepts for research involving human beings and was approved by the Brazilian Research Ethics Committee under opinion number 4,258,366. The participants received and electronically signed the Informed Consent Form. The entire data collection process contained no personal identification information to ensure anonymity.

## Results

This study received answers from 11,369 health professionals, representing all Brazilian regions. The participants were mostly female [9,075 (79.8%)] and, by region, there were 3,459 (30.4%) professionals from the Northeast and 3,228 (28.4%) from the Southeast, followed by the Midwest, North and South regions, with 2,015 (17.7%), 1,684 (14.8%) and 982 (8.6%), respectively.

Of the 11,369 professionals who participated in the study, 7,023 reported some type of skin lesion. The overall prevalence was 61.8% (95% CI: 60.9%-62.7%). Table 1 shows the frequency of skin lesions caused by using N95 respirators among health professionals.

As for the gender category, the results showed that the frequency of skin lesions among women is 5,769 (63.6%), followed by the male professionals with 1,254 (54.7%).

As for the region category, the results showed that the frequency of skin lesions among professionals from the Northeast region was 2,245 (64.9%), followed by the North region with 1,015 (60.3%), Midwest with 1,243 (61.7%), Southeast with 1,885 (58.4%), and South with 635 (64.7%).

As for the professional category, Nursing professionals reported the highest frequency of skin lesions caused by the use of N95 respirators (5,344 [61.9%]), as well as professionals who did not work in the ICU (4,956 [58.4%]), who did not work in a field hospital (4,627 [59.0%]), and who had no COVID-19 diagnosis (4,588 [59.8%]). In the univariate analysis, all the variables analyzed were associated with having skin lesions, as shown in Table 1. 

**Table 1:** Association between the occurrence of skin lesions caused by the use of N95 respirators among health professionals (n=11,369) and the demographic, professional and “use of Personal Protective Equipment” variables. Brazil, 2020

Variables	Skin lesion related to the use of N95 respirators		Total	p-value*	Prevalence Ratio	95% Confidence Interval
	Yes	No				
	n	n				
Gender						
Male	1,254 (54.7)	1,039 (45.3)	2,293		Reference	
Female	5,769 (63.6)	3,307 (36.4)	9,076	<0.01	1.162	(1.102;1.224)
Region						
Northeast	2,245 (64.9)	1,214 (35.1)	3,459		Reference	
North	1,015 (60.3)	669 (39.7)	1,684		0.929	(0.887;0.972)
Midwest	1,243 (61.7)	772 (38.3)	2,015	<0.01	0.950	(0.911;0.991)
Southeast	1,885 (58.4)	1,344 (41.6)	3,229		0.899	(0.865;0.934)
South	635 (64.7)	347 (35.3)	982		0.996	(0.917;1.018)
Professional Category						
Nursing professionals	5,344 (61.9)	3,283 (38.1)	8,627		Reference	
Physicians	816 (65.6)	428 (34.4)	1,244		1.059	(1.014;1.106)
Physiotherapists	439 (70.4)	185 (29.6)	624		1.136	(1.077;1.198)
Psychologists	59 (47.2)	66 (52.8)	125	<0.01	0.762	(0.632;0.918)
Speech Therapists	24 (55.8)	19 (44.2)	43		0.901	(0.690;1.716)
Occupational Therapists	18 (64.3)	10 (35.7)	28		1.038	(0.787;1.369)
Dentists	111 (48.3)	119 (51.7)	230		0.779	(0.681;0.891)
Other healthcare professionals	212 (47.3)	236 (52.7)	448		0.764	(0.692;0.843)
Worked in ICU†						
No	4,956 (58.4)	3,527(41.6)	8,483	<0.01	Reference	
Yes	2,067 (71.6)	819 (28.4)	2,886		1.226	(1.191;1.262)
Worked in a COVID-19 field hospital						
No	4,627 (59.0)	3,212 (41.0)	10,310	<0.01	Reference	
Yes	2,396 (67.9)	1,134 (32.1)	1,059		1.262	(1.226;1.299)
Infected by COVID-19						
No	4,588 (59.8)	3,082 (40.2)	7,670	<0.01	Reference	
Yes	2,435 (65.8)	1,264 (34.2)	3,699		1.100	(1.068;1.133)
Attended training or course on COVID-19						
Yes	5,127 (63.3)	2,973 (36.7)	8,100	<0.01	Reference	
Yes	1,896 (58.0)	1,373 (42.0)	3,269		0.916	(0.886;0.947)
The workplace provided sufficient PPE‡						
Yes	4,937 (59.4)	3,376 (40.6)	8,313		Reference	
No	374 (65.8)	194 (34.2)	568		1.109	(1.049;1.171)
Somehow	1,712 (68.8)	776 (31.2)	2,488	<0.01	1.159	(1.122;1.196)
The workplace provided good quality PPE‡						
Yes	3,537 (56.6)	2,708 (43.4)	6,245		Reference	
No	797 (68.7)	363 (31.3)	1,160	<0.01	1.213	(1.160;1.268)
Somehow	2,689 (67.8)	1,275 (32.2)	3,964		1.198	(1.162;1.235)

* p-value obtained from the chi-square test;

† Intensive Care Unit;

‡ Personal Protection Equipment

Regarding the type of skin lesions caused by the use of N95 respirators, the data showed that the professionals reported more than one type of skin change, with hyperemia as the most prevalent with 4,243 (60.4%), followed by dryness with 2,515 (35.8%), broken skin with 2,342 (33.3%), and blisters/bubbles with 613 (8.7%). As for the site of the injuries, the nose was the main place of occurrence identified by the participants, with 5,192 (73.9%) ( Table 2). 

**Table 2:** Association between the frequency of skin lesions due to the use of N95 respirators, and site of the injuries. Brazil, 2020

Variables	Have you had any skin changes related to the use of an N95 respirator?	
	Yes	No
	n (%)	n (%)
Type		
Hyperemia	4,243 (60.4)	2,780 (39.6)
Itching	2,086 (29.7)	4,937 (70.3)
Dryness	2,515 (35.8)	4,508 (64.2)
Broken Skin	2,342 (33.3)	4,681 (66.7)
Blisters/Bubbles	613 (8.7)	6,410 (91.3)
Not applicable	171 (2.4)	6,852 (97.6)
Site of the injury		
Nose	5,192 (73.9)	1,831 (26.1)
Cheek	4,180 (59.5)	2,843 (40.5)
Ear	1,719 (24.5)	5,304 (75.5)
Chin	1,500 (21.4)	5,523 (78.6)
None	67 (1.0)	6,956 (99.0)

The multivariable analysis of the values associated with skin lesions caused by the use of N95 respirators showed that female professionals are 1.203 times (95% CI: 1.154-1.255) more likely to have injuries when compared to their male counterparts. The professionals from the North, Midwest and Southeast regions were 0.923 times (95% CI: 0.879-0.970), 0.949 times (95% CI: 0.908-0.992) and 0.916 times (95% CI: 0.881-0.953) less likely to have skin lesions, respectively, when compared to the Northeast region ( Table 3). 

Regarding the professional category, the chances of skin lesions due to the use of N95 respirators among professional psychologists [Prevalence Ratio (PR)=0.805; 95% CI: 0.678-0.956] and dentists (PR=0.884; 95% CI: 0.788-0.992) were lower when compared to Nursing professionals. However, physicians and physiotherapists were more likely to have skin lesions when compared to Nursing professionals.

Regarding the workplace, working in the ICU increases the chances of having an injury: PR=1.203 (95% CI: 1.168-1.241). In addition, not working in a field hospital reduces the chances of having an injury: PR=0.889 (95% CI: 0.863-0.916).

Professionals with a positive COVID-19 diagnosis have an increased chance (PR=1.074; 95% CI: 1.042- 1.107) of having skin lesions resulting from the use of N95 respirators.

Goodness of fit of the model was tested with the Hosmer-Lemeshow test proposed by Hosmer and Lemeshow (2013). The results showed that fit of the model was good (p-value=0.3293) at a 95% confidence level.

**Table 3:** Prevalence ratio estimated from the regression model for skin injuries due to the use of N95 respirators among health professionals. Brazil, 2020

Variables	p-value*	Adjusted Prevalence Ratio	Confidence Interval (95%) for Prevalence Ratio	
Male (Reference)				
Female	<0.001	1.203	1.154	1.255
Northeast region (Reference)				
North region	<0.001	0.924	0.879	0.970
Midwest region	0.02	0.949	0.908	0.992
Southeast region	<0.001	0.916	0.881	0.953
South region	0.49	1.019	0.966	1.076
Nursing Professionals (Reference)				
Physicians	<0.001	1.111	1.069	1.161
Physiotherapists	0.02	1.082	1.019	1.148
Psychologists	0.004	0.805	0.678	0.956
Speech Therapists	0.38	0.897	0.692	1.162
Occupational Therapists	0.68	1.060	0.817	1.376
Dentists	0.02	0.884	0.788	0.992
Other	<0.001	0.837	0.766	0.914
Working in the ICU† (No) (Reference)				
Working in the ICU† (Yes)	<0.001	1.20	1.168	1.241
Working in a field hospital (Yes) (reference)				
Working in a field hospital (No)	<0.001	0.889	0.863	0.916
COVID-19 Diagnosis (No) (Reference)				
COVID-19 Diagnosis (Yes)	<0.001	1.074	1.042	1.107
Received training (No) (Reference)				
Received training (Yes)	<0.001	1.104	1.068	1.142
Insufficient PPE‡ provided by the workplace (reference)				
Sufficient PPE‡ provided by the workplace	0.64	0.982	0.909	1.060
Somewhat sufficient PPE‡ provided by the workplace	<0.001	1.071	0.993	1.155
Poor quality PPE‡ provided by the workplace (reference)				
Good quality PPE‡ provided by the workplace	<0.001	0.810	0.765	0.854
Somewhat good quality PPE‡ provided by the workplace	<0.001	0.942	0.888	1.000

* Chi-square Test;

† Intensive Care Unit;

‡ Individual Protection Equipment

**Figure 1:**
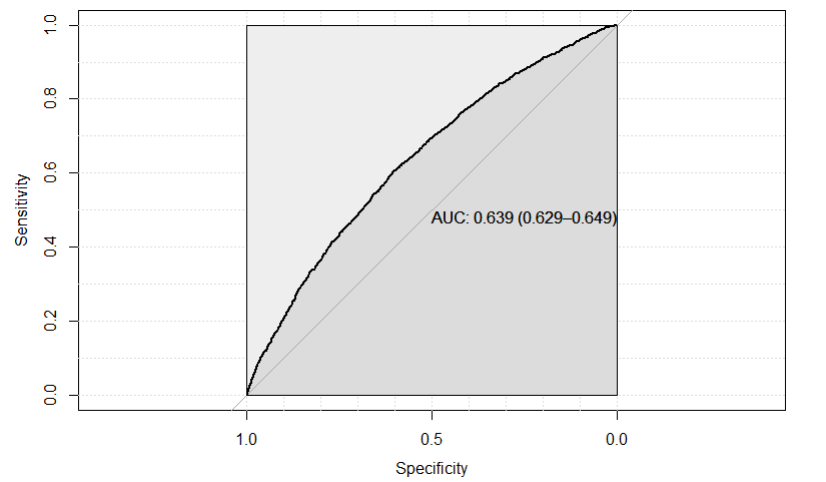
ROC curve of the adjusted model

Furthermore, in order to demonstrate the good quality of the model fit, the Receiver Operator Characteristic Curve (ROC) will also be presented ( Figure 1). The results show that the model’s accuracy (area under the curve) was equal to 0.639. The results also show that the model’s sensitivity was 0.873. This indicates that the model performs well to accurately estimate the risk of skin lesions caused by N95 respirators, considering the explanatory variables used to adjust the model. These results were obtained from the *confusion matrix* function from the *caret* package, of the Environment for Statistical Computing and Graphics R free software. 

## Discussion

This study was the first nation-wide and population-based survey on N95 respirators causing face skin lesion conducted with more than 10,000 healthcare professionals in all five major Brazilian regions. The findings can be highly generalizable and confirm the high prevalence rate of skin lesions caused by using N95 respirators.

The overall prevalence of skin lesions in this study was 61.8%. A similar study conducted in Brazil identified 69.4% prevalence of skin lesions among health professionals at the beginning of the COVID-19 pandemic ^( 8)^ . More studies should be added for a comparison ^( 13)^ . A study of 542 front-line healthcare workers in Hubei indicated a high prevalence rate of 97.0% skin damage caused by enhanced infection-prevention measures ^( 13)^ . This discrepancy would be greatly related to duration and frequency of each single use of N95 respirators. This assumption is justified and underpinned by the theory of pressure sores with interaction between time and pressure.

The professionals’ profile is comparable to the one found in previous studies that were similar in scope, in which the participating population is largely comprised by women in the Nursing category ^( 15, 21)^ . Consequently, female health professionals had higher prevalence values concerning the onset of skin changes due to the use of N95 respirators. 

A research study carried out in Hong Kong showed that, on average, N95 respirators are worn for more than five hours after each gowning procedure ^( 22)^ . Another study showed a significant relationship between using N95 respirators for more than four hours and the occurrence of skin lesions ^( 23)^ . In addition, N95 respirators present a higher risk of adverse skin reactions on the face when compared to other types of masks, such as cloth or surgical masks ^( 9)^ . 

To prevent the occurrence of skin lesions, a number of authors recommend that the N95 respirators must be removed from the face for 15 minutes every two hours, outside the environment of direct care for COVID-19 patients. If this is not feasible, the respirators should be removed from the face for at least five minutes every two hours ^( 9, 24)^ . 

The professionals reported more than one type of injury, with predominance of hyperemia, dryness and broken skin. This finding was consistent with studies on skin changes among health professionals on the front lines against COVID-19 ^( 8, 15)^ . 

Regarding the site where the skin lesions appeared, the nasal bridge was the most affected region. This is similar to what has been described in other studies ^( 22, 25)^ . A study of 526 front-line Chinese healthcare professionals in Hubei indicated that skin damage due to the use of N95 respirators and goggles on the nasal bridge (83.1%) and cheek (78.7%) were prevalent ^( 13)^ . N95 respirators directly compress the nose, which is an area that lacks subcutaneous tissue, and this causes skin changes in the presence of prolonged pressure, shearing and moisture ^( 9, 15)^ . Proper application and permanence time of N95 respirators are essential and must be communicated to each health professional to help prevent this morbidity ^( 22)^ . 

Using preventive coverings under N95 respirators was also found to minimize the occurrence of skin lesions ^( 14, 26)^ . Another study also showed that using a protection bundle, which included skin inspection, cleaning and hydration, as well as wearing a face mask with a skin protector, was associated with a reduction from 29% to 8% in the incidence of skin lesions ^( 27)^ . However, these add-on measures on tight-fitting respirators would be impaired by the high risk of leakage and render the fit test result invalid. 

Regarding the professional category, dentists were more likely to have skin lesions when compared to Nursing professionals. Dental surgeons were classified as a high-risk category for COVID-19 infection. Their physical proximity to the patient’s face, direct contact with mucous membranes and oral fluids, and frequent procedures that generate aerosols during their service to patients while wearing an N95 respirator favor the appearance of skin lesions ^( 28)^ . 

The fact that the occurrence of skin lesions associated with N95 respirators was self-reported represents a study limitation. However, as the participants are health professionals and these skin lesions are easily identified, the information was considered reliable.

Thus, the study contributed to increasing the knowledge about aspects that are relevant to skin lesions in health professionals due to the use of N95 masks. In addition, identifying factors associated with the occurrence of this event is essential for the development of measures to prevent these injuries in health professionals.

## Conclusion

This study showed high prevalence of skin lesions in health professionals who reported using N95 respirators in their practice. As for the types of skin lesions caused by use of these devices, there was predominance of hyperemia, followed by dryness, with the nasal bridge as the main site. The prevalence of skin lesions caused by the use of N95 respirators was associated with female gender, region of the country, professional category, workplace, training, COVID-19 diagnosis, and availability of sufficient good-quality Personal Protective Equipment.

The results showed the need to adopt strategies to protect and prevent skin damage among the health professionals who are at the front line of care for COVID-19 patients while using N95 respirators.
